# Accurate Intervertebral Disc Segmentation Approach Based on Deep Learning

**DOI:** 10.3390/diagnostics14020191

**Published:** 2024-01-16

**Authors:** Yu-Kai Cheng, Chih-Lung Lin, Yi-Chi Huang, Guo-Shiang Lin, Zhen-You Lian, Cheng-Hung Chuang

**Affiliations:** 1 Department of Neurosurgery, China Medical University Hospital, Taichung 404, Taiwan; master3743@gmail.com; 2 Department of Neurosurgery, Asia University Hospital, Taichung 413, Taiwan; jefflin0529@gmail.com; 3 Department of Occupational Therapy, Asia University, Taichung 413, Taiwan; 4 Department of Radiology, Asia University Hospital, Taichung 413, Taiwan; yichi710103@gmail.com; 5 Department of Computer Science and Information Engineering, National Chin-Yi University of Technology, Taichung 411, Taiwan; gslin@ncut.edu.tw; 6 Department of Artificial Intelligence and Computer Engineering, National Chin-Yi University of Technology, Taichung 411, Taiwan; s00098122@gmail.com

**Keywords:** deep learning, u-net, distance transformation, intervertebral disc segmentation

## Abstract

Automatically segmenting specific tissues or structures from medical images is a straightforward task for deep learning models. However, identifying a few specific objects from a group of similar targets can be a challenging task. This study focuses on the segmentation of certain specific intervertebral discs from lateral spine images acquired from an MRI scanner. In this research, an approach is proposed that utilizes MultiResUNet models and employs saliency maps for target intervertebral disc segmentation. First, a sub-image cropping method is used to separate the target discs. This method uses MultiResUNet to predict the saliency maps of target discs and crop sub-images for easier segmentation. Then, MultiResUNet is used to segment the target discs in these sub-images. The distance maps of the segmented discs are then calculated and combined with their original image for data augmentation to predict the remaining target discs. The training set and test set use 2674 and 308 MRI images, respectively. Experimental results demonstrate that the proposed method significantly enhances segmentation accuracy to about 98%. The performance of this approach highlights its effectiveness in segmenting specific intervertebral discs from closely similar discs.

## 1. Introduction

Low back pain, or lumbar pain, stands as one of the most prevalent issues faced by individuals and ranks as the fifth leading cause of medical consultations. It impacts the daily lives of at least 7.6% to 37% of patients [1,2,3], with 10% of patients experiencing chronic pain and mobility challenges [1]. There exists a close association between low back pain and intervertebral disc degeneration. In fact, degeneration of disc tissue begins earlier than other muscle and bone tissue and often occurs without any symptoms. According to the literature, the onset of initial intervertebral disc degeneration may commence as early as adolescence. About 20% of young people show mild symptoms [4], and the incidence gradually increases with age. Degenerative disc disease affects 10% of the male population at the age of 50 and up to 50% of the male population at the age of 70. In some reports, degenerative disc disease may be present in as many as 90% of individuals [5].

The human spine consists of the cervical spine (7 vertebrae, C1–C7), thoracic spine (12 vertebrae, T1–T12), lumbar spine (5 vertebrae, L1–L5), and sacral spine (5 vertebrae, S1–S5) [6]. The primary function of the spine is to support the weight of the entire body. Among the vertebral bones of the spine, the largest lumbar vertebrae, comprising 5 vertebrae (L1–L5), bear a significant portion of the upper body weight [7]. Intervertebral discs are fibrous tissues located between two vertebrae and are named after the vertebrae they connect. In clinical practice, neurosurgeons or orthopedic surgeons can analyze and diagnose the degree of intervertebral disc degeneration by measuring the height of the lumbar intervertebral disc and observing the condition of the intervertebral disc [7,8]. Obtaining spinal imaging through techniques such as computed tomography (CT) or magnetic resonance imaging (MRI) is crucial for diagnosing degenerative disc disease [9]. Among these, MRI images provide a deeper understanding of the biochemical and structural characteristics of tissues and can detect the fat and water content of intervertebral discs and vertebrae [10]. Therefore, this study utilizes an image database that includes MRI scans as experimental materials.

To diagnose intervertebral disc degeneration, neurosurgeons or orthopedic surgeons typically need to analyze and compare three specific discs: L1/L2, L4/L5, and L5/S1. This is because the discs L4/L5 and L5/S1, located below the lumbar spine, are more prone to degeneration, while the disc L1/L2, situated above the lumbar spine, is less susceptible to degeneration [11]. Generally, neurosurgeons or orthopedic surgeons manually mark these three discs for diagnosis. If carried out solely manually, such a workflow can be labor-intensive and time-consuming. Therefore, there is a need for a system that can automatically segment and identify the specific intervertebral disc specified by the physician.

Recently, convolutional neural networks (CNNs) have ushered in significant advancements within the field of image segmentation and recognition, particularly within the domain of medical imagery. Consequently, deep learning models have gained extensive adoption in the medical sector. For instance, Ronneberger et al. introduced the U-Net framework [12], which has demonstrated remarkable efficiency in segmenting neuronal structures within electron microscopy stacks, leveraging pre-existing annotated data. Another noteworthy approach, proposed by Kayalibay et al. [13], employs a CNN-based method employing three-dimensional filters to proficiently segment hand and brain MRI images. Oktai et al. introduced the attention-driven U-Net model [14], designed for medical imaging segmentation, with the unique capability to autonomously adapt its focus to target structures of diverse sizes and shapes. Singh et al. [15] proposed a graph network-based module called latent graph attention (LGA) to incorporate global context into the existing CNN architectures. LGA uses a network of locally connected graphs to propagate information spatially, helping to establish a semantically coherent relationship between any two spatially distant points, thereby achieving better object segmentation.

Furthermore, Ibtehaz et al. introduced the MultiResUNet model [16], which extends the performance of the U-Net model to better address multimodal medical image segmentation. Lastly, Lou et al. devised the DC-UNet model [17], a modification of the classic U-Net framework that has exhibited noticeable enhancements in performance. Wang et al. [18] proposed a mixed transformer module for simultaneous inter- and intra-affinity learning and constructed a U-shaped model named Mixed Transformer U-Net (MT-UNet) for accurate medical image segmentation. Chen et al. [19] proposed a transformer-based attention guidance network called TransAttUnet, which can enhance the representation of multi-scale contextual information to generate discriminative features and effectively alleviate the loss of fine details caused by convolutional layer stacking and continuous sampling operations, ultimately improving the segmentation quality of medical images.

Several deep learning models have emerged in the scientific literature, focusing on the segmentation of MRI images depicting intervertebral discs [20,21,22,23,24,25,26,27,28,29]. For instance, Wang et al. [20] introduced a convolutional architecture based on the 3D U-Net, designed for the segmentation of 66 intervertebral discs within multimodal MRI images. In a separate study, Vania et al. [21] devised a multistage optimization approach, utilizing mask-RCNN for the segmentation of intervertebral discs across 263 patients with T1 and T2 images. Meanwhile, Das et al. [22] proposed a novel region-to-image matching network model for the identification and segmentation of intervertebral discs within 24 multimodal MRI images derived from 16 subjects. Their model showcased an average identification accuracy of 92.5%. Li et al. [23] designed a semi-supervised semantic segmentation network for spine images based on conditional adversarial neural networks combined with U-Net and Tversky loss to solve the segmentation problem and obtained an accuracy of 89.8%. Mushtaq et al. [24] used YOLOv5 for vertebral localization and segmentation and achieved a mean average precision of 97.5% from MRI scans of 514 subjects. The cropped images derived from YOLOv5 bounding boxes undergo processing through HED U-Net, a hybrid framework encompassing both segmentation and edge detection, to acquire segmented vertebrae along with their corresponding edges. Then, Harris corner detector was applied to obtain the corners of the desired vertebrae to determine LLAs (lumbar lordotic angles) and LSAs (lumbosacral angles) for lumbar lordosis diagnosis. The diagnostic accuracy rate was 74.5%.

Hess et al. [25] utilized convolutional neural networks to segment vertebral bodies, intervertebral discs, and paraspinous muscles in T1-weighted MRI images from a dataset of 206 MRI exams. Their results show that the segmentation masks and associated metrics exhibited high similarity between human- and computer-generated methods, with Dice coefficients of 0.77. Wang et al. [26] proposed a deep learning model based on a 3D Deeplab V3+ network to automatically segment multiple structures from MRI images at the L4/5 level. The deep learning model obtained an average precision of 89.9% from a total of 50 participants who had undergone a 3T MRI with T2-3D-space sequences. Wang et al. [27] presented a modified U-Net network by adding multi-scale blocks and residuals for spinal segmentation and achieved an average segmentation accuracy of over 88% from 210 adult spinal MRI images. Altun et al. [28] used U-Net-based methods to segment the lumbar spinal stenosis region. They found that the highest segmentation success among 1560 images was obtained in the ResUNet model, with a 0.93 DICE score. Lu et al. [29] proposed a model called ConvMixEst and Muti-Attention Unet (CAM-Unet), which combined multilayer perceptron with the attentional mechanisms of inverted variational attention and dilated gated attention and obtained a precision of 94.09%. Nonetheless, it is worth noting that many of these approaches tend to segment all intervertebral discs within an image as opposed to targeting some specific discs, and they also grapple with the challenge of a limited amount of original image data.

This study aims to perform segmentation based on the intervertebral discs specified by clinical doctors for the diagnosis of disc degeneration. Therefore, the segmentation targets are specified according to the clinical doctors’ requirements, focusing on three different intervertebral discs: L1/L2, L4/L5, and L5/S1. Approximately 3000 lateral spine MRI images were collected for this research to alleviate the limitations caused by insufficient image data. Several different models, including U-Net [12], CNN-based [13], Attention U-Net [14], and MultiResUNet [16], were employed to segment the three specific intervertebral discs, revealing numerous instances of segmentation errors. Among them, U-Net and MultiResUNet have the highest segmentation accuracy, which is 76.6% and 83.4%, respectively. However, none of the models achieved an average intersection-over-union (IoU) value exceeding 72.3%. These inaccuracies include redundant segmentations of intervertebral discs and erroneous segmentations of non-specific intervertebral discs. This difficulty arises from the highly trivial and similar nature of intervertebral disc tissue, as well as significant variations between slices, making accurate intervertebral disc segmentation challenging [29].

Since MultiResUNet shows higher accuracy compared to U-Net, the literature [30] proposes a two-stage method based on the MultiResUNet framework. This method divides the target intervertebral disc into upper and lower parts. In the first stage, MultiResUNet was used to segment the lower discs, and the segmented discs were used to generate distance maps. In the second stage, the original image and distance maps were combined and used to segment the upper disc. The method achieved an accuracy of 93.8% and a mean IoU metric of 77.1%. While the two-stage MultiResUNet [30] has significantly improved segmentation accuracy, there is still a gap before its practical clinical application. Clinicians aim to achieve higher accuracy rates. Therefore, this study proposes a more accurate segmentation approach based on MultiResUNet and saliency maps. Experimental results demonstrate that this method can enhance the segmentation accuracy of specific intervertebral discs to about 98%, with the mean IoU metric remaining at 77%.

The main contributions of this study can be summarized as follows:

- The proposed method can identify some specific intervertebral discs from a group of similar ones.
- The saliency map prediction algorithm improves the accuracy of cropping the target intervertebral disc.
- The sub-image cropping method reduces the amount of data required to be processed, speeds up computation time, and potentially improves prediction accuracy.
- The data-augmented segmentation method uses distance maps to help improve prediction accuracy.

## 2. Materials and Methods

### 2.1. Study Design

This study is retrospective. The goal of this research is to create a high-precision intervertebral disc segmentation model based on deep learning. Compared with traditional image processing or manual methods, deep learning methods have the advantages of high accuracy, speed, and saving operation time and labor. This segmentation model can replace the work of manually segmenting the intervertebral discs and can specify segmentation targets at the clinician’s request. Current work focuses on segmenting three different intervertebral discs: L1/L2, L4/L5, and L5/S1. These three intervertebral discs can help clinicians diagnose intervertebral disc degeneration.

### 2.2. Data and Ground Truth

The experimental images used in this study were a total of 2982 de-identified spinal lateral images collected from MRI scanners at Asia University Hospital in Taichung, Taiwan, from August 2016 to July 2020. When we acquired these deidentified images, there was no interaction with the patients. That is, this study did not include any interaction or intervention with human subjects or any access to identifiable private information. Therefore, this study complied with the ethical standards of the institutional and national committees on human experimentation and was performed in accordance with the guidelines of the Declaration of Helsinki. 

The image data used midsagittal slices of the lumbar spine and were converted into 512 × 512-pixel images. It mainly includes three parts: the thoracic, lumbar, and sacral spines. The images were manually labeled by a physician with the three intervertebral discs between the lumbar spine and sacrum, namely L1/L2, L4/L5, and L5/S1. During the collection process, cases with anatomic abnormalities (such as lumbar sacralization or sacrolumbarization) that lacked these three intervertebral discs were excluded. This was due to the small number of images for these cases of anatomical abnormalities, and this study did not classify any spinal disorders. We expect to accurately measure the size and heterogeneity of segmental discs so that future studies on the correlation between discs and spinal degeneration can be performed. As long as the three intervertebral discs of the image (L1/L2, L4/L5, and L5/S1) could be manually segmented by the physician, the images were collected in our experimental data.

**Figure 1:**
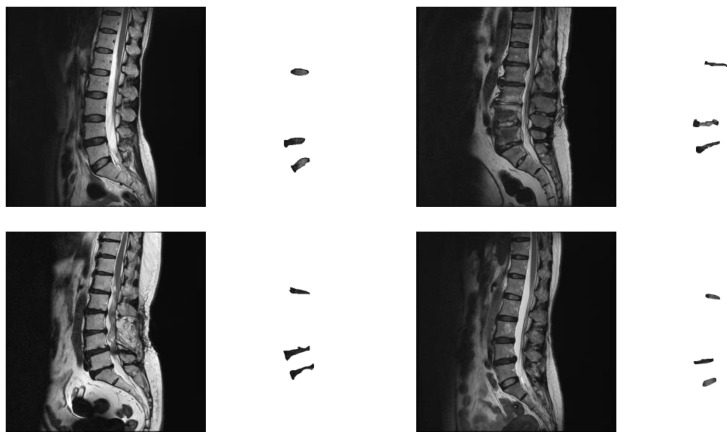
Experimental image samples and the corresponding manually labeled mask.

The number of experimental images is calculated based on the sample size estimated by the confidence interval [31]. If a 95% confidence interval is set and a standard deviation of 0.01 and a margin of error of 0.04% are used, then the most conservative sample size can be calculated, which is approximately 2400 images. The number of collected images, about 3000 images, exceeded the most conservative sample size required. The experimental images were randomly divided into a training set and a test set, which are 2674 and 308 images, respectively. Figure 1 shows some experimental image samples and corresponding manually labeled standard masks.

### 2.3. Model

**Figure 2:**
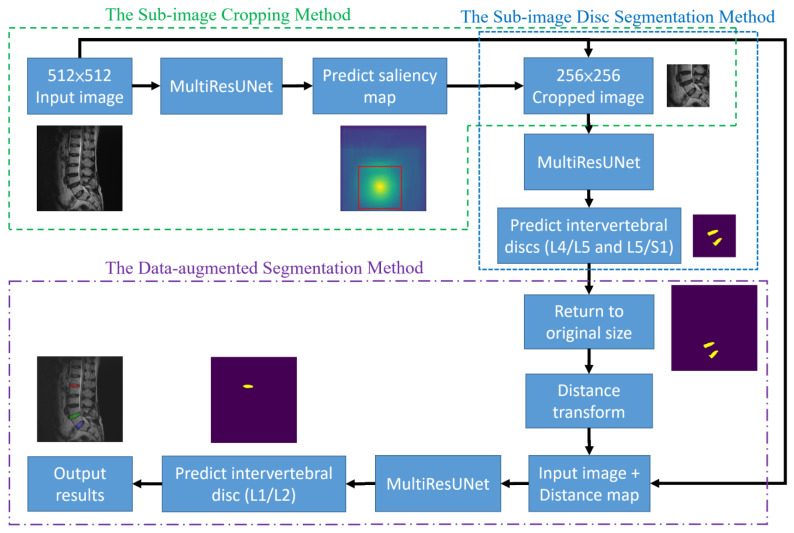
The flow chart of the proposed approach.

This section presents the details of the proposed approach for the segmentation of target intervertebral discs based on deep learning. The overall structure of the proposed approach is shown in Figure 2. The arrow here indicates the direction of the steps and has no special meaning. The structure consists of three methods, namely the sub-image cropping method, the sub-image disc segmentation method, and the data-augmented segmentation method. The sub-image cropping method predicts the original image through MultiResUNet to obtain the saliency map. This saliency map highlights the location of the center points of the target intervertebral discs so that sub-images can be cropped to improve prediction accuracy. The sub-image disc segmentation method uses MultiResUNet to predict the sub-image to obtain the target discs. The data-augmented segmentation method calculates the distance map of the segmented discs as augmented data and then predicts the remaining target discs through MultiResUNet. These three methods will be explained in detail in Section 2.3.1, Section 2.3.2 and Section 2.3.3.

The training and testing algorithms of the proposed method are described as follows.

Training algorithm:

1. For each of the 2674 training images, load its standard masks.
2. Compute the saliency map from standard masks.
3. Use training images and saliency maps to train MultiResUNet_Model_1.
4. Cropping training images and their standard masks to sub-images.
5. Use cropped images and standard masks to train MultiResUNet_Model_2.
6. Compute distance maps from standard masks.
7. Combine original images and distance maps.
8. Use original images + distance maps, and standard masks to train MultiResUNet_Model_3.

Testing algorithm:

1. For each of the 308 testing images
2. Input testing images to MultiResUNet_Model_1 to predict saliency maps.
3. Cropping testing images to sub-images by saliency maps.
4. Input cropped testing images to MultiResUNet_Model_2 to predict masks.
5. Return the predicted mask sub-images to the original image size.
6. Compute distance maps from the predicted masks.
7. Combine original images with distance maps.
8. Input original images + distance maps to MultiResUNet_Model_3 to predict masks.
9. Integrate all the predicted discs together.

#### 2.3.1. The Sub-Image Cropping Method

The initial step of the proposed approach is to extract sub-images containing the target intervertebral discs (L4/L5 and L5/S1). The original image size is 512 × 512, while the sub-image size is 256 × 256. The size of the sub-image is half the length and half the width of the original image and needs to completely contain each individual disc. The scanning algorithm is to use a block of size 256 × 256 to sequentially crop the original image from left to right and bottom to top. The sequentially cropped 256 × 256 sub-images are used to train MultiResUNet to successfully predict intervertebral discs. During testing, the sequentially cropped 256 × 256 sub-images are input into MultiResUNet to predict the intervertebral discs. If the predicted disc area changes in the sub-image of the sequence, it means that the disc is not completely included in the sub-image. This method continues until there is no change in the predicted disc area in the sub-image. At this time, it means that the intervertebral disc is completely included in the sub-image.

However, the sub-images extracted from the scanning algorithm may encompass various locally cropped intervertebral discs. This results in poor prediction performance for MultiResUNet due to significant changes in disc shape. Therefore, a saliency map prediction algorithm that uses distance transforms to predict the positions of intervertebral discs is proposed in this study. The saliency map is an image that highlights the location of the center points of the target intervertebral discs. The center point of the target intervertebral discs is calculated from known disc masks in the training set. Subsequently, the distance transforms calculate *D_i_* from each pixel coordinate point (*x_i_*, *y_i_*) to the center point (*x*_0_, *y*_0_), where *i* represents the sequence number of the pixel in the image. The calculation formula for *D_i_* is as follows:

(1) $$D_{i}=1-\frac{{\left(\sqrt{{\left(x_{i}-x_{0}\right)}^{2}+{\left(y_{i}-y_{0}\right)}^{2}}\right)}^{n}}{\max({\left(\sqrt{{\left(x_{i}-x_{0}\right)}^{2}+{\left(y_{i}-y_{0}\right)}^{2}}\right)}^{n})}$$

where *n* is the power of distance.

**Figure 3:**
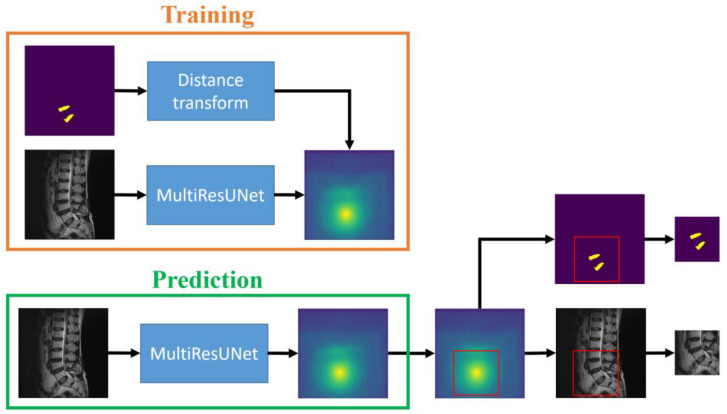
The training and prediction flow chart of the saliency map prediction algorithm in the sub-image cropping method.

From Equation (1), it is evident that pixels closer to the center point yield larger values. The MultiResUNet is employed to train each original image to its corresponding saliency map. After completing the training, this network can be used to predict the saliency maps of the test set images. By utilizing the predicted saliency maps, it is possible to estimate the center points of the target intervertebral discs and subsequently crop sub-images from the original image. The training and prediction flow chart of the saliency map prediction algorithm in the sub-image cropping method is shown in Figure 3. The workflow is depicted in the flowchart, as illustrated in Figure 2, following the steps from left to right at the top of the figure. During the training process, saliency maps are obtained through distance transformation using standard masks. The original images are then trained by MultiResUNet to obtain the saliency maps. The prediction process is to input the test images into MultiResUNet to obtain the predicted saliency maps. Then, the locations of the sub-images can be extracted from the saliency maps.

#### 2.3.2. The Sub-Image Disc Segmentation Method

**Figure 4:**
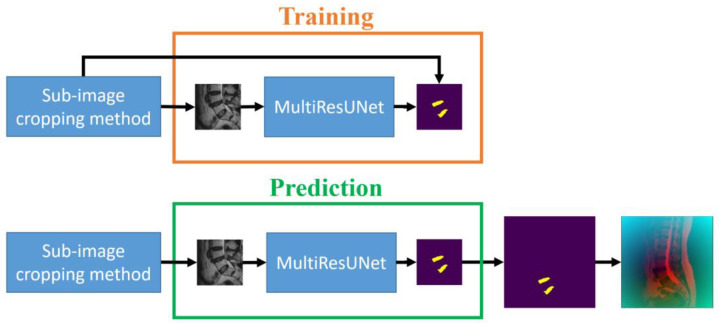
The training and prediction flow chart for sub-image disc segmentation method.

The second step of our approach aims to segment the target intervertebral discs (L4/L5 and L5/S1) following the initial prediction, which already allowed for the cropping of sub-images containing the target intervertebral discs. Utilizing these 256 × 256 sub-images along with the corresponding intervertebral disc masks from the training set, MultiResUNet is employed to train each sub-image to its corresponding intervertebral disc mask. Upon completing the training, this can be used to predict the target intervertebral discs. The sub-image size constitutes one-quarter of the original image size. The main advantages of this approach are the reduction in data volume, acceleration of computation time, and potential enhancement of prediction accuracy. However, it is essential to restore the image size to 512 × 512 pixels following the prediction, using the preserved original positions of the sub-images. The training and prediction flow chart of the MultiResUNet for the sub-image disc segmentation method is shown in Figure 4. The flowchart for this part of the process is outlined in Figure 2, proceeding from top to middle on the right side of the figure. During the training process, sub-images and their masks are obtained from the sub-image cropping method. The sub-images are then trained by MultiResUNet to obtain the corresponding masks. The prediction process is to input the test sub-images into MultiResUNet to obtain the predicted masks. Then, the predicted masks are restored to their original image size.

#### 2.3.3. The Data-Augmented Segmentation Method

**Figure 5:**
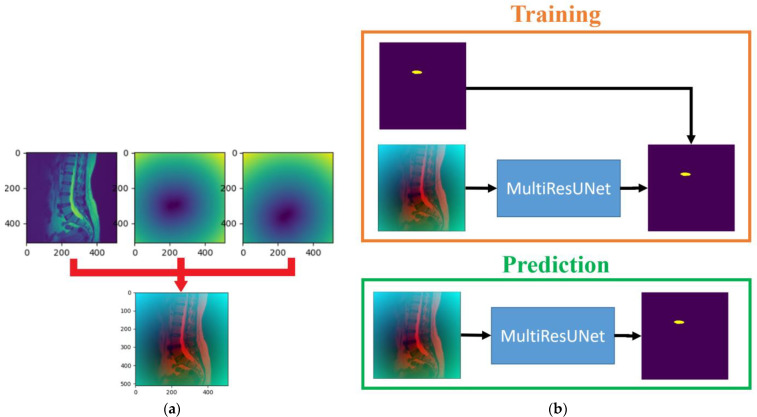
(**a**) An example of an original image augmented with the distance maps. (**b**) The training and prediction flow chart of the data-augmented segmentation method.

The third step of the proposed approach is to segment the upper target intervertebral disc (L1/L2) using a data-augmented segmentation method. Upon examining the shape of intervertebral discs, it is evident that the L1/L2 intervertebral disc bears a striking resemblance to its adjacent intervertebral discs. If the original image and the upper intervertebral disc mask were directly used to train the MultiResUNet model, segmentation errors often occurred during prediction, leading to the detection of neighboring intervertebral discs. Since the positions of the lower target intervertebral discs can be determined following the second prediction, distance maps can be employed here as auxiliary information to predict the upper target intervertebral disc. The distance transformation is used to calculate the distance maps for the lower target intervertebral discs. Distance values are zero at the center point of the intervertebral disc, and they increase as coordinate points move farther from the center. After normalization, these distance values form a distance map. Two distance maps are obtained from the two target intervertebral discs for data augmentation. The training dataset for this step consists of original images augmented with distance maps. Figure 5a shows an example of an original image augmented with the distance maps. The MultiResUNet is employed to train the image data to its corresponding target intervertebral disc mask. Following the completion of training, it can be utilized to predict the final target intervertebral disc. The final output results from the combination of the predicted upper intervertebral disc with the two lower intervertebral discs. The training and prediction flow chart of the data-augmented segmentation method is shown in Figure 5b. The flowchart for this final stage is illustrated in Figure 2, proceeding from right to left at the bottom of the figure.

### 2.4. Evaluation

The experiments in this study use a training data set of 2674 images and a test data set of 308 images to evaluate the optimal power *n* of distance in Equation (1), the performance of the saliency map prediction algorithm, and the performance of the proposed method. The number of training epochs in the MultiResUNet is set to 500. The images for the experiments have a total of three intervertebral discs marked manually by physicians, i.e., L1/L2, L4/L5, and L5/S1. These experiments use a test data set consisting of 308 images to evaluate the accuracy of segmentation. Firstly, the segmented images must be categorized into two classes: one for images with correct predictions and the other for images with incorrect predictions. The calculation of accuracy involves dividing the number of images with correct predictions by the total number of images in the test dataset. Assuming the total number of images in the test dataset is denoted as T, then T = 308. 

Next, the evaluation of correct predictions is divided into two steps. The first step is to assess whether the predicted number of discs is correct. Connected-component labeling is employed to calculate the predicted number of intervertebral discs. If the predicted number of intervertebral discs equals 1, it is considered correct; otherwise, it is deemed incorrect. The second step is to evaluate whether the predicted disc area is correct. The evaluation of segmentation accuracy is commonly assessed using the Intersection over Union (IoU) metric [12,16]. The IoU measures the overlap ratio between the segmented region and the ground truth region, which is the intersection of these regions divided by their union. In the ideal case, where there is a perfect overlap, the IoU ratio equals 1. Generally, an IoU ratio greater than or equal to 0.5 is considered acceptable, indicating correct detection. In this study, the threshold for correct detection is set to 0.7, aiming to enhance the overlap between the predicted area and the ground truth area. Assuming the predicted area is denoted as $R_{j}^{k}$ and the ground truth area is denoted as $S_{j}^{k}$, where *j* represents the index of the predicted image (*j* = 1, 2, …, *T*) and *k* represents the index of the intervertebral disc (*k* = 1, 2, 3), the IoU calculation formula for the *k*-th intervertebral disc in the *j*-th predicted image is as follows:

(2) $$IoU_{j}^{k}=\frac{R_{j}^{k}\cap S_{j}^{k}}{R_{j}^{k}\cup S_{j}^{k}}$$

Next, a flag *f* is set to indicate the correctness or incorrectness of the status. If the predictions for all three intervertebral discs in the *j*-th predicted image are correct, the flag for this predicted image is set to 1; otherwise, it is set to 0. The formula for the flag is as follows:

(3) $$f_{j}=\left\{\begin{array}{l} 1,\;\mathrm{where}\;\forall k:\;IoU_{j}^{k}\geq 0.7\\ 0,\;\mathrm{otherwise}\; \end{array}\right.$$

Assuming the accuracy is denoted as *A* and the number of correctly predicted images is *B*, then the formula for calculating accuracy is as follows:

(4) $$A=\frac{B}{T}=\frac{1}{T}\sum_{j=1}^{T}f_{j}$$

The mean IoU is also used to assess the overlap ratio between the predicted intervertebral discs and the standard ground truth masks. Although the test dataset is annotated with manually segmented ground truth masks by physicians, these ground truth masks serve as a criterion for evaluation. The formula for calculating the mean IoU of a specific disc, i.e., L1/L2, L4/L5, or L5/S1, is as follows:

(5) $$\bar{IoU^{k}}=\frac{1}{T}\sum_{j=1}^{T}IoU_{j}^{k}$$

where *k* represents the index of the intervertebral disc (*k* = 1, 2, 3). The experiment sets the number of predicted intervertebral discs to 3. The formula for the mean IoU of different methods is also modified as follows:

(6) $$\bar{IoU}=\frac{1}{3T}\sum_{j=1}^{T}\sum_{k=1}^{3}IoU_{j}^{k}$$

The structural similarity index measure (SSIM) between the standard and predicted intervertebral discs is also provided for different methods. If the SSIM is closer to 1, it means the structures are more similar; otherwise, they are not similar.

## 3. Results

The experimental equipment is shown as follows: The hardware used was an Intel^®^ Core™ i9-9900K processor and 64GB of memory, with a NVIDIA GeForce GTX 2080 11GB graphics card. The software used was the Windows 10 64-bit operating system, PyTorch 1.8.1, CUDA Toolkit 10.2, and Python 3.7.6. The total training time, including evaluation time, is about 75 h. The total test time for all 308 images is 676.5 s, which is about 2.2 s for one image.

### 3.1. The Evaluation of Power of Distance

**Figure 6:**
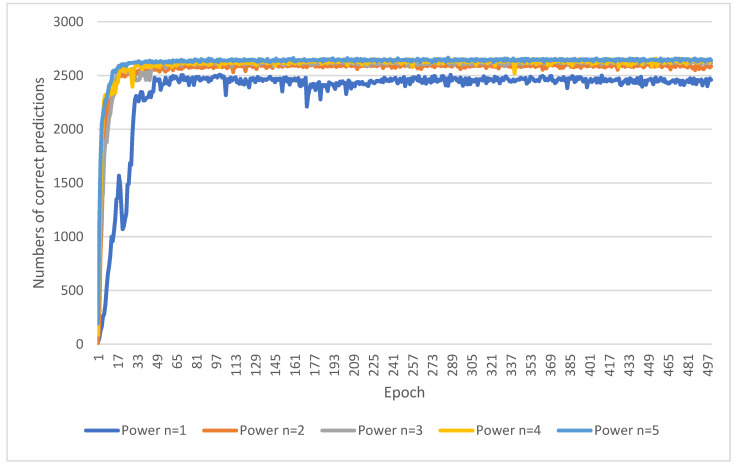
The numbers of correct predictions in the 500 training epoch using different powers of distance in the saliency map prediction algorithm.

From the sub-image cropping method in Section 2.3.1, the saliency map prediction algorithm needs to evaluate the optimal power *n* of distance in Equation (1). The flow chart of the saliency map prediction algorithm is shown in Figure 3. The power *n* of distance is set from 1 to 5. The center point coordinates (*x*, *y*) of the resultant saliency map are compared with the standard center point coordinates (*x*_0_, *y*_0_). The comparison method is to calculate the error values of the *x* and *y* coordinates, i.e., |*x* − *x*_0_| + |*y* − *y*_0_|. If the error value is less than 10, the prediction is considered correct. On the contrary, if the error value is greater than or equal to 10, it is regarded as a wrong prediction. Figure 6 shows the numbers of correct predictions in 500 training epochs when using different powers of distance in the saliency map prediction algorithm. It is obvious that within the first 50 training epochs, the network has not yet stabilized, resulting in a smaller number of correct predictions. After 50 training epochs, the curve oscillates steadily and slightly. However, the oscillation of the curve with a power of one is more serious. Table 1 intercepts the training process in the 51–500 epochs to compare the performance of different powers of distance. Experiments have found that as the power gradually increases, the number of correct predictions also approximately gradually increases. The best results in Table 1 are the data with a power of five.

Next, the experiment must evaluate the performance of the saliency map prediction algorithm on the test data set of 308 images. The experiment was evaluated based on the training process range of 51–500 epochs. A total of 450 tests are performed, and the number of correct predictions can be obtained for each test. At the same time, the error distance between the center point coordinates of the predicted saliency map and the standard center point coordinates is calculated during the test process. Table 2 shows the average number and percentage of correct predictions made during the test process, as well as the average error distance. In Table 2, it is obvious that the results when the power is between 3 and 5 have the same and maximum number of correct predictions. However, the results with a power of three have the smallest error distance of 8.7. Therefore, the power *n* of distance in Equation (1) in the saliency map prediction algorithm is set to three.

### 3.2. The Evaluation of Sub-Image Cropping Method

In the sub-image cropping method, two algorithms have been proposed: the scanning algorithm and the saliency map prediction algorithm. This experiment is required to evaluate and compare these two algorithms. The experimental design is to use these two algorithms to perform sub-image cropping of individual intervertebral discs. The cropped sub-images are then used for intervertebral disc segmentation. The performance of sub-image cropping methods is compared by the number of images with correctly segmented discs.

**Figure 7:**
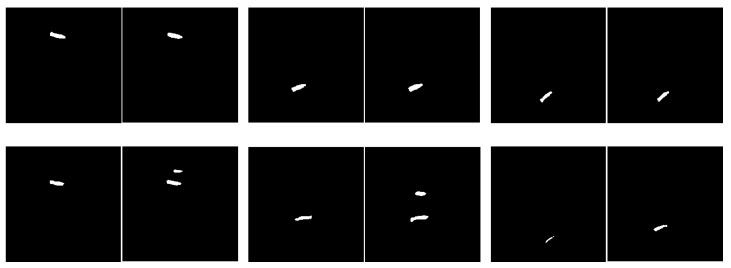
Correct versus incorrect sampling images of individual disc segmentation. The upper row represents correct segmentation, and the lower row represents incorrect segmentation. The left side of each pair of images is the standard mask, and the right side is the predicted result.

Table 3 shows the performance comparison of scanning and saliency map prediction algorithms for single-disc segmentation. The experimental steps are to perform the sub-image cropping method and the sub-image disc segmentation method on a single intervertebral disc and restore the result to the original image size. From the perspective of the number and accuracy of correctly predicted images, it is obvious that the saliency map prediction algorithm is better than the scanning algorithm. The L1/L2 intervertebral disc has lower values of the number and accuracy of correctly predicted images because this disc is very similar to its adjacent discs. Its segmentation is more challenging. The mean IoU is also roughly higher than 70%, except for the L1/L2 intervertebral disc. Figure 7 shows the correct and incorrect sampling images of a single intervertebral disc segmentation. Most of the segmentation errors occur when the wrong disc is found or the IoU value is lower than 0.7.

### 3.3. The Evaluation of Proposed Method

**Figure 8:**
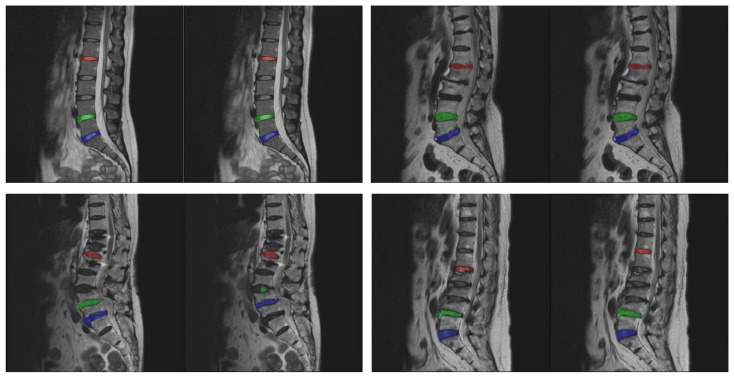
Sampling results of the proposed approach. The upper row shows the correct results, and the lower row shows the error results. The left side of each pair of images is the standard mask, and the right side is the predicted result.

Next, experiments are conducted to evaluate the accuracy of various different methods. The approach proposed in this study needs to be compared with five other different models to evaluate the performance of different methods, including U-Net [12], CNN-based [13], Attention U-Net [14], Multi-ResUNet [16], and the two-stage Multi-ResUNet model [30]. A comparison between the proposed method and the experimental data of the aforementioned five models is shown in Table 4. The steps of the proposed method are to perform the sub-image cropping method and the sub-image disc segmentation method on the two lower target discs (L4/L5 and L5/S1), and to perform the data-augmented segmentation method on the upper disc (L1/L2). The comparison results show that with a confidence interval of 95%, the accuracy of the proposed approach reaches 97.7 ± 1.6%, the mean IoU is 77.0 ± 0.4%, and the SSIM is 0.6916 ± 0.0115, surpassing the performance of the other five models. Figure 8 shows the results of the proposed approach. The upper row shows the correct results, and the lower row shows the error results. In each pair of images, the left image is the standard mask, and the right one is the prediction result. The three target intervertebral discs (L1/L2, L4/L5, and L5/S1) in the top row of Figure 8 are accurately segmented. In the bottom row of Figure 8, the case on the left is an error in the segmentation of the two lower target intervertebral discs (L4/L5 and L5/S1), and the case on the right is an error in the segmentation of the upper intervertebral disc (L1/L2).

## 4. Discussion

In general, most deep learning models can easily achieve the segmentation of multiple similar target regions. Particularly, deep learning models based on U-Net exhibit excellent performance in medical image segmentation. However, when the segmentation task involves localizing specific target regions among them, the prediction accuracy significantly deteriorates. It is easier to find the wrong intervertebral disc when segmenting one single disc, as shown in the lower row of images in Figure 7. For instance, when attempting to predict the intervertebral disc L1/L2, one may end up with neighboring discs such as T12/L1 or L2/L3. It can be clearly observed from the results that the accuracy rate in Table 3 is lower than that of the proposed method in Table 4. As evident from the results in Table 4, directly segmenting the three intervertebral discs with U-Net-based models does not yield high accuracy; e.g., the accuracy of U-Net [12] and MultiResUNet [16] is 76.6% and 83.4%, respectively, although their mean IoU does indeed exceed 70%. Furthermore, some U-Net-based models perform worse, such as CNN-based [13] and Attention U-Net [14], which achieve accuracy scores of only 13.3% and 30.8%, respectively, with the mean IoU approaching 60%. This indicates that these methods are not applicable to the questions in this study.

In Table 4, the accuracy of two-stage MultiResUNet [30] is improved to 93.8 ± 2.4%, with a mean IoU of 77.1 ± 1.0% exceeding the threshold of 70%. The SSIM index also increases from 0.6696 ± 0.0155 for U-Net to 0.6859 ± 0.0134 for MultiResUNet. Although the accuracy of the two-stage MultiResUNet [30] has improved to approximately ninety percent, there is still room for improvement. We conducted a detailed investigation into the two-stage MultiResUNet method and found that many errors occurred during the first stage, where the lower intervertebral discs L4/L5 and L5/S1 were cropped and predicted. These errors included the misprediction of intervertebral discs such as L3/L4 and L4/L5. The primary reason for these errors is the high similarity between adjacent intervertebral discs and the presence of many incomplete intervertebral discs during the training of MultiResUNet. This is due to the use of traditional image processing methods for cropping sub-images, which leads to imprecise cropping of the target intervertebral discs.

To address this issue, this study proposes the use of a deep learning model, the saliency map prediction algorithm, to crop the target intervertebral discs. For a detailed description of the method, please refer to Section 2.3. The original images can predict saliency maps, which are essentially distance maps, through MultiResUNet. By predicting these saliency maps, we can obtain the center points of the target intervertebral discs and then crop sub-images with complete intervertebral discs. In Table 4, the accuracy of the proposed method is improved to 97.7 ± 1.6%, with a mean IoU of 77.0 ± 0.4%, which exceeds the threshold of 70%, and the SSIM index is also improved to 0.6916 ± 0.0115.

## 5. Conclusions

This study presents a precision segmentation approach based on deep learning models that can accurately predict specific target intervertebral discs required for clinical diagnosis. In the first step, the approach employs MultiResUNet as the prediction model and uses a sub-image cropping method based on a saliency map prediction algorithm. In the saliency map prediction algorithm, it predicts the saliency maps of the target intervertebral discs. These saliency maps are then used to crop sub-images containing the target intervertebral discs. This sub-image is one-quarter of the original image, which reduces the amount of data required to be processed, speeds up computation time, and potentially improves prediction accuracy. In the second step, a MultiResUNet is used to predict the target intervertebral discs in the sub-images. After obtaining target intervertebral discs, the distance transform is used to generate distance maps for data-augmented segmentation in the third step. The original images are combined with distance maps and used to train MultiResUNet to segment the remaining target intervertebral discs accurately. Therefore, all target intervertebral discs (L1/L2, L4/L5, and L5/S1) can be predicted with precision.

Experimental results demonstrate a significant improvement in segmentation accuracy using our proposed approach. In the evaluation, the segmentation accuracy is approximately 98%, and the mean IoU is about 77%. Compared to the results of MultiResUNet [16], the accuracy and mean IoU of the proposed approach have increased by approximately 15% and 5%, respectively. In comparison with the two-stage MultiResUNet [30], the accuracy of the proposed approach has improved by about 4%.

Future research directions include a comparative analysis with the highly regarded U2-Net and the currently employed MultiResUNet. Furthermore, it is hoped that this research method can find practical application in clinical diagnosis by providing neurosurgeons with a convenient means to analyze and compare specific target intervertebral discs, thereby reducing the cost of manual segmentation. The approach can also serve as preprocessing for any intervertebral disc segmentation and identification in future clinical studies and be applied to the segmentation of a few specific targets among multiple similar targets.

## Figures and Tables

**Table 1:** Performance comparison of the different powers of distance in the training process of the saliency map prediction algorithm (ranges from 51 to 500 epochs).

Power of Distance	Maximum No. of Correct Predictions	Minimum No. of Correct Predictions	Average No. of Correct Predictions	Percentage of Correct Predictions
*n* = 1	2507	2210	2450	91.6%
*n* = 2	2613	2532	2589	96.8%
*n* = 3	2647	2571	2618	97.9%
*n* = 4	2657	2519	2629	98.3%
*n* = 5	2666	2616	2643	98.9%

**Table 2:** Performance comparison of the different powers of distance in the testing process of the saliency map prediction algorithm (a total of 450 tests for each power of distance).

Power of Distance	Average No. of Correct Predictions	Percentage of Correct Predictions	Average Error Values |*x* − *x*_0_| + |*y* − *y*_0_|
*n* = 1	196	63.6%	14.1
*n* = 2	228	74.0%	14.9
*n* = 3	269	87.3%	8.7
*n* = 4	250	81.2%	14.3
*n* = 5	269	87.3%	9.2

**Table 3:** Performance comparison of the scanning and saliency map prediction algorithms for individual intervertebral disc segmentation.

Sub-Image Cropping Method	Intervertebral Disc	Number of Correctly Predicted Images (*B*)	Number of Error Predicted Images (*T*–*B*)	Accuracy (*A*)	Mean IoU
Scanning	L1/L2	203	105	65.9%	56.2%
	L4/L5	287	21	93.2%	75.0%
	L5/S1	283	25	91.9%	75.0%
Saliency Map Prediction	L1/L2	274	34	89.0%	67.1%
	L4/L5	292	16	94.8%	72.2%
	L5/S1	295	13	95.8%	74.8%

**Table 4:** Performance comparison of the proposed method and other methods (with a confidence interval of 95%).

Model	Number of Correctly Predicted Images (*B*)	Number of Error Predicted Images (*T*–*B*)	Accuracy (*A*)	Mean IoU	SSIM
U-Net [12]	236	72	76.6 ± 0.9%	71.9 ± 0.2%	0.6696 ± 0.0155
CNN-based [13]	41	267	13.3 ± 0.3%	55.5 ± 0.2%	0.5421 ± 0.0139
Attention U-Net [14]	95	213	30.8 ± 0.9%	58.2 ± 0.4%	0.6830 ± 0.0155
MultiResUNet [16]	257	51	83.4 ± 0.3%	72.3 ± 1.0%	0.6859 ± 0.0134
Two-stage [30]	289	19	93.8 ± 2.4%	77.1 ± 1.0%	0.6901 ± 0.0138
Proposed Method	301	7	97.7 ± 1.6%	77.0 ± 0.4%	0.6916 ± 0.0115

## Data Availability

Restrictions apply to the availability of these data. Data were obtained from the Department of Radiology at Asia University Hospital and are available from Yi-Chi Huang with the permission of Asia University Hospital.
